# Clinical Relevance of Impaired Physiological Assessment After Percutaneous Coronary Intervention: A Meta-analysis

**DOI:** 10.1016/j.jscai.2022.100448

**Published:** 2022-09-08

**Authors:** Alexander M. Griffioen, Stijn C.H. van den Oord, Steven Teerenstra, Peter Damman, Niels van Royen, Robert Jan M. van Geuns

**Affiliations:** a Department of Cardiology, Radboud University Medical Center, Nijmegen, the Netherlands; b Department of Cardiology, Rijnstate Hospital, Arnhem, the Netherlands; c Section Biostatistics, Department for Health Evidence, Radboud University Medical Center, Nijmegen, the Netherlands

**Keywords:** fractional flow reserve, instantaneous wave-free ratio, percutaneous coronary intervention, physiological assessment, quantitative flow ratio, major adverse cardiac events

## Abstract

**Background:**

Despite the optimal angiographic result of percutaneous coronary intervention (PCI), residual disease at the site of the culprit lesion can lead to major adverse cardiac events. Post-PCI physiological assessment can identify residual stenosis. This meta-analysis aims to investigate data of studies examining post-PCI physiological assessment in relation to long-term outcomes.

**Methods:**

Studies were included in the meta-analysis after performing a systematic literature search on July 1, 2022. The primary end point was the incidence of major adverse cardiac events, vessel-orientated cardiac events, or target vessel failure.

**Results:**

Low post-PCI fractional flow reserve, reported in 7 studies with fractional flow reserve cutoff values between 0.84 and 0.90, including 4017 patients, was associated with an increased rate of the primary end point (hazard ratio [HR], 2.06; 95% CI, 1.37-3.08). One study reported about impaired post-PCI instantaneous wave-free ratio with instantaneous wave-free ratio cutoff value of 0.95 in relation to major adverse cardiac events, showing a significant association (HR, 3.38; 95% CI, 0.99-11.6; *P* = .04). Low post-PCI quantitative flow ratio, reported in 3 studies with quantitative flow ratio cutoff value between 0.89 and 0.91, including 1181 patients, was associated with an increased rate of vessel-orientated cardiac events (HR, 3.01; 95% CI, 2.10-4.32). Combining data of all modalities, impaired physiological assessment showed an increased rate of the primary end point (HR, 2.32; 95% CI, 1.71-3.16) and secondary end points, including death (HR, 1.41; 95% CI, 1.04-1.89), myocardial infarction (HR, 2.70; 95% CI, 1.34-5.42) and target vessel revascularization (HR, 2.88; 95% CI, 1.91-4.35).

**Conclusions:**

Impaired post-PCI physiological assessment is associated with increased adverse cardiac events and individual end points, including death, myocardial infarction, and target vessel revascularization. Therefore, prospective studies are awaited on whether physiology-based optimization of PCI results in better clinical outcomes.

## HIGHLIGHTS

• Adverse cardiac events are related with impaired physiological assessment.
• Impaired physiological assessment is related with death, myocardial infarction, and target vessel revascularization.
• The modality applied for physiological assessment does not influence results.

## Central Illustration

**Figure:**
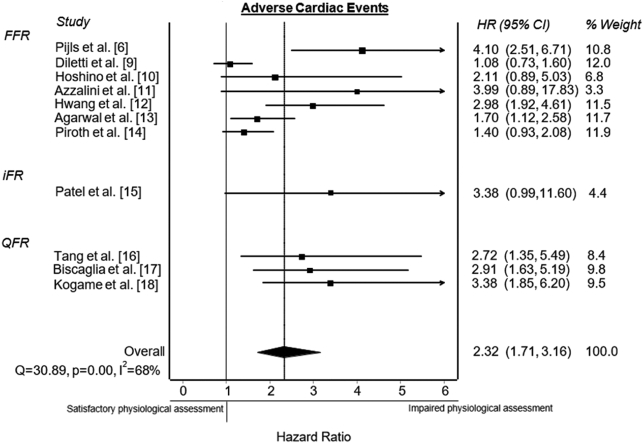
**Post-PCIphysiologicalassessment andadversecardiacevents.** Forest plots of hazard ratios (HR) of post-PCI physiological assessment and adverse cardiac events, including MACE, VOCE and TVF, defined according to the included studies. Markers represent point estimates of HRs. Marker size represents study weight. Horizontal bars indicate 95% CIs. FFR, fractional flow reserve; iFR, instantaneous wave-free ratio; MACE, major adverse cardiac events; PCI, percutaneous coronary intervention; QFR, quantitative flow ratio; TVF, target vessel failure; VOCE, vessel-orientated cardiac events.

## Introduction

Physiology-guided percutaneous coronary intervention (PCI) is important to guide clinicians in the decision-making for coronary revascularization. Nonhyperemic pressure ratios, including resting full-cycle ratio and instantaneous wave-free ratio (iFR), and hyperemic pressure ratios, including fractional flow reserve (FFR), are used for physiological assessment. Lesions with resting full-cycle ratio value <0.89, iFR value <0.89, or FFR value <0.80 can cause myocardial ischemia.^1^^,^^2^ Using this FFR cutoff value for PCI compared with angiography-guided PCI results in a reduced rate of major adverse cardiovascular events (MACE).^3^ Unfortunately, despite the optimal angiographic result of PCI, the incidence of MACE after PCI remains high. In an all-comers follow-up study after PCI, MACE was reported in 19% to 32% of the patients at 2 years of follow-up, depending on the used stent type during PCI.^4^ In addition, Stone et al^5^ demonstrated that in up to 13% of all patients, major cardiovascular events after PCI are caused by residual or recurrent disease at the site of the culprit lesion.

Physiological assessment of lesions with the angiographically optimal result can be used to identify residual stenosis. Pijls et al^6^ already demonstrated 2 decades ago that higher FFR values after PCI are associated with decreased rates of MACE. In the following decades, several studies reported on the association between FFR after PCI and adverse cardiac events. More recently, alternative modalities such as iFR and QFR have been studied in relation to adverse cardiac events, providing additional data. The studies, however, are diverse, performed with different technologies and providing outcomes with varying significance. Additionally, as a result of the small sample size, the power of several studies is low.

The goal of this systematic review and meta-analysis is to provide an overview of data with a minimum follow-up of 6 months on post-PCI physiological assessment and long-term outcomes in patients with acute coronary syndromes and chronic coronary syndrome and to provide a pooled analysis of post-PCI FFR, iFR and QFR data in relation with long-term outcomes.

## Methods

### Search strategy

This study was performed using the Meta-analysis of Observational Studies in Epidemiology guidelines.^7^ Systematic search of the literature was performed in PubMed, Embase, and Web of Science on July 1, 2022.

A combination of the following keywords for FFR was used: “Fractional Flow Reserve, myocardial,” “FFR,” “Coronary Circulation,” “Treatment Outcome,” “Percutaneous Coronary Intervention.” A literature search on iFR and QFR was performed using the following keywords: “Post PCI iFR,” “instantaneous wave-free ratio,” “Post PCI QFR,” and “Quantitative Flow Ratio.” A literature search was preferably performed using Medical Subject Headings terms. Duplicates, animal studies, non-English language articles, systematic reviews, and meta-analyses were erased. In addition, abstracts only and unpublished studies were not included. Two independent reviewers (A.M.G and S.C.H.vdO) screened and identified all appropriate titles and abstracts. A review of the reference lists of these studies was performed to find additional relevant studies. After a full-text review, studies that reported data on the measurement of post-PCI FFR, iFR, and QFR and long-term outcomes in patients with acute coronary syndrome and chronic coronary syndrome were selected for inclusion in this systematic review and meta-analysis. If multiple studies from the same cohort were identified, selection was made based on the sufficiency of the data. The Newcastle-Ottawa Scale was used to assess the quality of the selected nonrandomized studies.

### Data collection

Data of interest from the included studies were extracted by 2 independent reviewers (A.M.G. and S.C.H.vdO). General data included information on study design, in- and exclusion criteria, sample size, and demographic characteristics. Data on the procedural characteristics included information on angiographic data and pre- and post-PCI FFR, iFR, and QFR data. Follow-up data included information on the follow-up duration, indicated to be for at least 6 months, and data on the used end points, defined as MACE (defined as a composite of cardiac death, myocardial infarction [MI], and any revascularization), vessel-orientated cardiac events (VOCE) (defined as cardiovascular death, vessel-related [spontaneous] MI and [ischemia-driven] target vessel revascularization [TVR]) and target vessel failure (TVF) (defined as a composite of cardiac death, target vessel MI, and clinically-driven TVR), according to the individual study.

### Statistical analysis

Statistical analysis was performed using Microsoft Excel 2016 (Microsoft Corporation) and MetaXL version 5.3 (EpiGear, www.epigear.com). The primary end point of this study was the incidence of MACE, defined according to the individual study. If MACE was not reported, comparable definitions, including VOCE and TVF, were used as the primary end point. Secondary end points included the incidence of death, MI, and TVR. Meta-analysis was performed on a combination of the hazard ratios (HRs) for MACE, VOCE, or TVF in relation to post-PCI FFR, iFR, or QFR, depending on which end point was reported by the included studies. To avoid any influence of confounding factors, HRs derived from the multivariate analysis were used to calculate an overall HR. If HRs derived from the multivariate analysis were not available, HRs derived from univariate analysis were used. If only survival curves with *P* values were present, HRs were reconstructed using WebPlotDigitizer version 4.5 (https://automeris.io/WebPlotDigitizer) and the method from Parmar et al^8^ If 2 or more studies provided sufficient data, an overall HR was calculated. *P* < .05 was considered statistically significant. HRs were plotted in a forest plot. Random effect models were used to calculate summary estimates and to construct forest plots. To assess the heterogeneity among the studies, the Q test and I^2^ index were used. Publication bias was assessed by funnel plot.

## Results

### Post-PCI FFR

**Figure 1:**
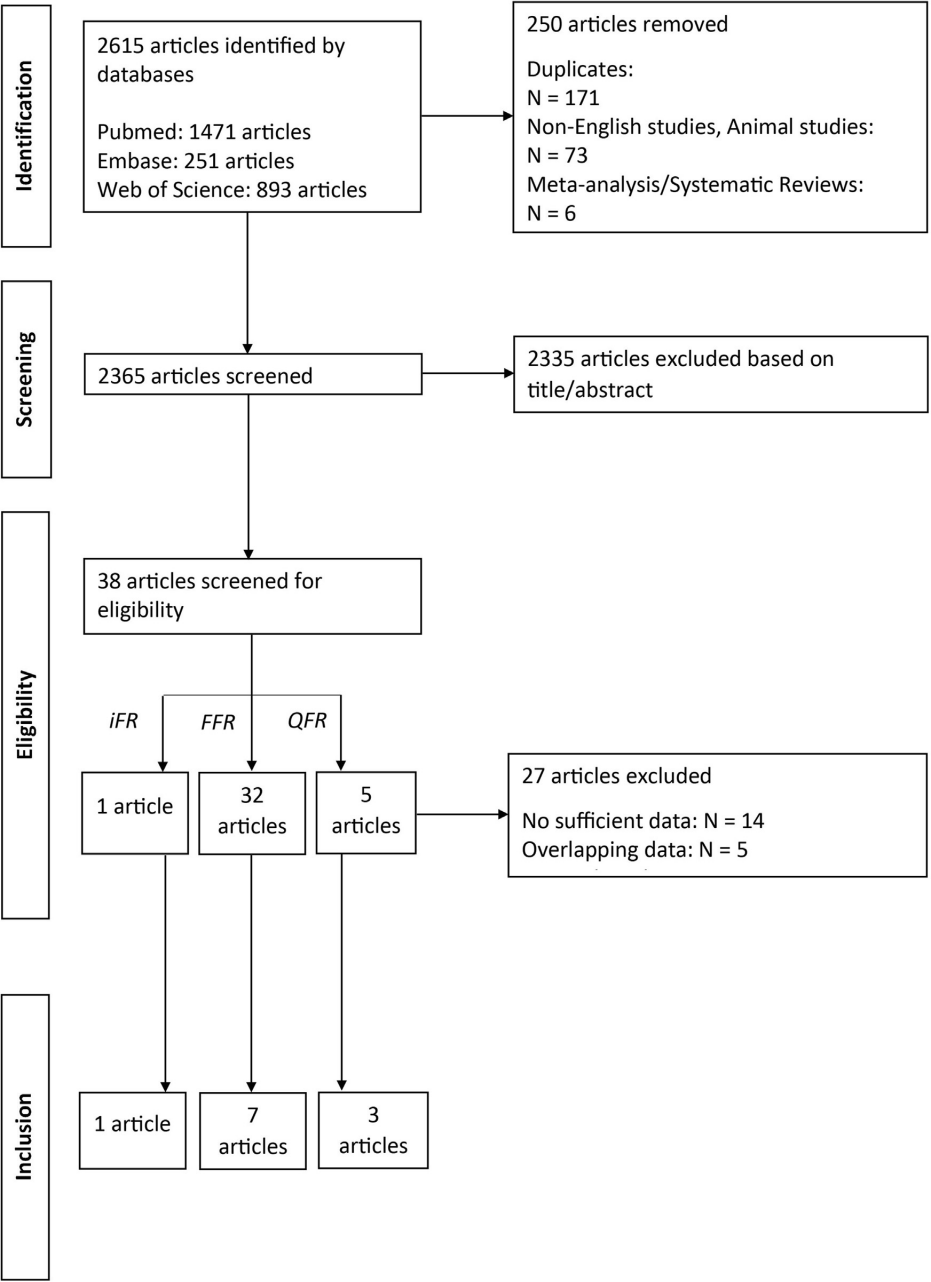
**Literature search strategy on post-percutaneous coronary interventionphysiological assessment.** FFR, fractional flow reserve; iFR, instantaneous wave-free ratio; QFR, quantitative flow ratio.

**Figure 2:**
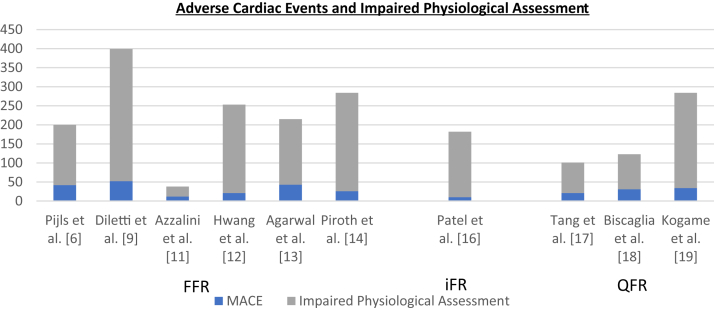
**Ratio between adverse cardiac events and impaired physiological assessment.** The plot of absolute numbers of adverse cardiac events related to the number of impaired physiological assessments categorized for each study. FFR, fractional flow reserve; iFR, instantaneous wave-free ratio; MACE, major adverse cardiovascular events; QFR, quantitative flow ratio.

**Figure 3:**
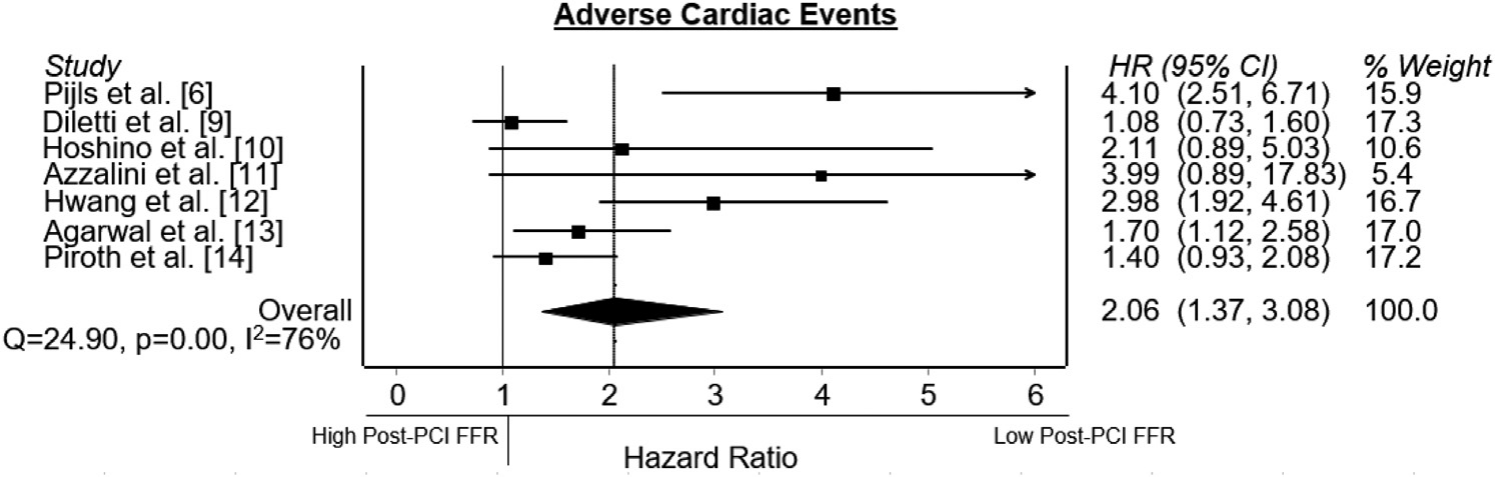
**Post-PCI FFR and adverse cardiac events.** Forest plots of hazard ratios (HR) of post-PCI FFR and adverse cardiac events, including MACE, VOCE, and TVF, are defined according to the included studies. Markers represent point estimates of HRs. Marker size represents study weight. Horizontal bars indicate 95% CIs. FFR, fractional flow reserve; MACE, major adverse cardiovascular events; PCI, percutaneous coronary intervention; TVF, target vessel failure; VOCE, vessel-orientated cardiac events.

A total of 32 articles were identified reporting data on post-PCI FFR and long-term outcomes (Supplemental Table S1). A total of 18 articles were excluded because no sufficient data were reported or data was overlapping. Data in 9 articles were incomplete. The corresponding authors of these articles were contacted and requested to supply additional data. Additional data was provided for 2 articles. Eventually, data in 7 articles^6^^,^^9^, ^10^, ^11^, ^12^, ^13^, ^14^ were sufficient to be included in the meta-analysis (Figure 1). Study quality assessment demonstrated good quality for 6 studies (Supplemental Table S2). All 7 studies reported data on MACE, VOCE, or TVF. An overview of the study characteristics is provided in Table 1, including the ratio between acute and chronic coronary syndromes and the cutoff value for impaired post-PCI physiological assessment. The studies included 65 to 959 patients (4017 patients in total). The median follow-up time was 23 months (6-31 months). Patient and procedural characteristics are summarized in Table 2 and Table 3. MACE was defined and reported by 5 studies (Table 1), with a total of 2543 patients. The prevalence of MACE over the 5 studies varied from 10.2% to 23.3%. VOCE was described in 1 study (n = 639).^14^ TVF was described in 1 study (n = 835) as well.^12^ The ratio between absolute numbers of adverse cardiac events and impaired post-PCI FFR is depicted in Figure 2. The meta-analysis showed a significant association between a low post-PCI FFR and the primary end point (HR, 2.06; 95% CI, 1.37-3.08) (Figure 3). The heterogeneity of the studies was 76%, suggesting that substantial between-study heterogeneity may be present in the studies, according to the Cochrane guideline.^15^

**Table 1:** Characteristics of the included studies.

Reference, year	Design	Indication for PCI	PCI technique	Cutoff value for impaired physiological assessment	Definition of MACE/VOCE/TVF
Post-PCI FFR					
Pijls et al,^6^ 2002	Prospective, observational	All comers	Stent (type NA)	PE: 0.90 SE: 0.90	MACE (composite of death, MI, and TVR, either by PCI or CABG)
Diletti et al,^9^ 2021	Prospective, observational	¼ stable angina, ¾ ACS	Stent (type NA)	PE: 0.90 SE: 0.90	MACE (composite of cardiac death, MI, any revascularization)
Hoshino et al,^10^ 2019	Retrospective, observational	All comers, only LAD	DES	PE: 0.86 SE: NA	MACE (VOCE [cardiovascular death, vessel-related spontaneous myocardial infarction, ischemia-driven LAD revascularization] and nontarget VOCE)
Azzalini et al,^11^ 2019	Prospective, observational	⅔ stable angina, ¼ ACS	DES/BRS/DEB	PE: 0.90 SE: NA	MACE (composite of all-cause death, nonfatal MI, and TVR)
Hwang et al,^12^ 2019	Prospective, observational	½ stable angina, ½ ACS	Second-generation DES	PE: 0.84 SE: 0.84	TVF (composite of cardiac death, TV-MI, and clinically-driven TVR)
Agarwal et al,^13^ 2016	Retrospective, observational	⅔ stable angina, ⅓ ACS	DES/BMS	PE: 0.86 SE: 0.87 (death), 0.85 (TVR)	MACE (defined as a composite of death, myocardial infarction, and TVR)
Piroth et al,^14^ 2017	Retrospective	All comers	DES	PE: 0.88 SE: 0.88	VOCE (composite of vessel-related cardiovascular death, vessel-related spontaneous MI, and ischemia-driven TVR)
Post-PCI iFR					
Patel et al,^16^ 2022	Prospective, observational	½ stable angina, ½ ACS	DES	PE: 0.95 SE: NA	MACE (composite of MI [defined as periprocedural or spontaneous], TVR or clinically-driven TVR, and cardiovascular death)
Post-PCI QFR					
Tang et al,^17^ 2020	Retrospective, observational	STEMI	Stent (type NA)	PE: 0.91 SE: 0.91	VOCE (composite of vessel-related cardiovascular death, vessel-related MI, and TVR)
Biscaglia et al,^18^ 2019	Prospective, observational	⅓ Stable angina, ⅔ NSTEMI	DES	PE: 0.89 SE: 0.89	VOCE (composite of vessel-related cardiovascular death, vessel-related MI, and ischemia-driven ​TVR)
Kogame et al,^19^ 2019	Retrospective, observational	¾ stable angina ¼ unstable angina	DES	PE: 0.91 SE: 0.91	VOCE (composite of vessel-related cardiac death, vessel-related MI, and TVR)

ACS, acute coronary syndrome; BMS, bare metal stent; BRS, bioresorbable scaffold; CABG, coronary artery bypass grafting; DEB, drug-eluting balloon; DES, drug-eluting stent; FFR, fractional flow reserve; iFR, instantaneous wave-free ratio; LAD, left anterior descending; MACE, major adverse cardiac events; MI, myocardial infarction; NA, not applicable; NSTEMI, non-ST-elevation myocardial infarction; PCI, percutaneous coronary intervention; PE, primary end point; QFR, quantitative flow ratio; SE, secondary end point; STEMI, ST-elevation myocardial infarction; TVF, target vessel failure; TV-MI, target vessel myocardial infarction; TVR, target vessel revascularization; VOCE, vessel-orientated cardiac events.

**Table 2:** Patient characteristics of the included studies.

	Pijls et al^6^ (N = 744)	Diletti et al^9^ (N = 959)	Hoshino et al^10^ (N = 201)	Azzalini et al^11^ (N = 65)	Hwang et al^12^ (N = 835)	Agarwal et al^13^ (N = 574)	Piroth et al^14^ (N = 639)	Patel et al^16^ (N = 467)	Tang et al^17^ (N = 186)	Biscaglia et al^18^ (N = 602)	Kogame et al^19^ (n = 393)
Age, y	62 ± 11	64.6 ± 11.8	67.0 (60.0-72.0)	68.9 ± 9.3	64.0 (56.0-72.0)	64.0 ± 9	64.0 ± 10	67.0 (60.0-73.0)	63.1 ± 11.7	68 (60-77)	66.6 ± 9.8
Male	NA	725 (76)	160 (80)	59 (91)	651 (78.0)	560 (98)	506 (79)	355 (76)	140 (75.3)	443 (74)	364 (92.6)
Hypertension	352 (51)	515 (54)	145 (72)	51 (79)	512 (61.3)	536 (93)	435 (68)	356 (76.2)	115 (61.8)	444 (74)	295 (75.4)
Diabetes	171 (23)	191 (20)	92 (46)	21 (32)	295 (35.3)	267 (45)	159 (25)	154 (33.0)	65 (34.9)	139 (23)	116 (29.7)
Dyslipidemia	416 (61)	451 (45)	121 (60)	41 (63)	422 (50.7)	488 (85)	472 (74)	324 (69.4)	35 (18.8)	336 (56)	297 (77.1)
Smoking	324 (47)	499 (52)	63 (31)	6 (9)	230 (27.6)	193 (34)	152 (24)	75 (16.1)	126 (67.7)	114 (19)	56 (14.7)
Prior MI	NA	203 (21)	NA	20 (31)	65 (7.8)	147 (26)	284 (37)	127 (27.2)	6 (3.2)	133 (22)	48 (12.3)
Prior PCI	NA	264 (28)	43 (21)	42 (65)	NA	NA	145 (23)	208 (44.5)	8 (4.3)	147 (24)	NA
Multivessel disease	NA	NA	21 (10)	NA	NA	321 (56)	NA	NA	NA	125 (21)	NA
Stable angina	NA	304 (32)	NA	43 (66)	452 (54.1)	NA	NA	197 (42.2%)	NA	200 (33)	272 (69.4)
Unstable angina/NSTEMI	NA	367 (38)	NA	15 (23)	383 (45.9)	NA	NA	223 (47.8)	NA	402 (67)	98 (25.0)
STEMI	NA	329 (34)	NA	NA	NA	NA	NA	NA	186 (100)	NA	NA
Follow-up, mo	6	22	24	12	23	31	24	12	24	21	24

Values are mean ± SD, median (IQR), or n (%).

FFR, fractional flow reserve; iFR, instantaneous wave-free ratio; MI, myocardial infarction; NA, not available; NSTEMI, non-ST-elevation myocardial infarction; PCI, percutaneous coronary intervention; QFR, quantitative flow ratio; STEMI, ST-elevation myocardial infarction.

**Table 3:** Procedural characteristics of the included studies.

	Pijls et al^6^ (N = 744 vessels)	Diletti et al^9^ (N = 1165 vessels)	Hoshino et al^10^ (N = 201 vessels)	Azzalini et al^11^ (N = 65 vessels)	Hwang et al^12^ (N = 835 vessels)	Agarwal et al^13^ (N = 664 vessels)	Piroth et al^14^ (N = 838 vessels)	Tang et al^17^ (N = 415 vessels)	Biscaglia et al^18^ (N = 751 vessels)	Kogame et al^19^ (N = 771 vessels)
Target vessel										
LM	NA	19 (2)	NA	3 (5)	NA	NA	NA	NA	NA	NA
LAD	372 (52)	593 (51)	201 (100)	47 (72)	603 (72.2)	295 (44)	433 (52)	169 (40.7)	356 (48)	352 (45.7)
RCx	122 (17)	211 (18)	NA	6 (9)	90 (10.8)	NA	188 (22)	106 (25.5)	184 (24)	243 (31.5)
RCA	219 (31)	331 (28)	NA	9 (14)	142 (17.0)	NA	217 (26)	140 (33.7)	211 (28)	176 (22.8)
Stents										
Stent length, mm	17.3 ± 6.4	23.0 (15-36)	28.0 (22.0-38.0)	37.9 ± 25.4	25.1 (18.2-32.1)	20.2 ± 8.4	23 ± 13	35.6 ± 16.8	30 (24-32)	42.6 ± 24.2
Stent diameter, mm	3.25 ± 0.42	3.0 (3.0-4.0)	3.3 (3.0-3.5)	NA	NA	2.87 ± 0.43	NA	2.97 ± 0.39	3 (3-3.5)	NA
No. of stents	NA	1.4 ± 0.6	NA	1.5 ± 1.0	NA	NA	1.3 ± 0.6	1.29 ± 0.52	1 (1-2)	1.7 ± 0.9
Physiological assessment										
Baseline	0.61 ± 0.17	NA	0.73 (0.64-0.77)	NA	0.71 (0.62-0.76)	0.65 ± 0.14	0.63 ± 0.14	NA	NA	0.67 ± 0.18
Post-PCI	0.92 ± 0.07	0.91 (0.07)	0.86 (0.82-0.89)	0.87 ± 0.07	0.86 (0.82-0.91)	0.89 ± 0.06	0.90 ± 0.06	0.94 ± 0.09	0.97 (0.92-0.99)	0.91 ± 0.07

Values are mean ± SD, median (IQR), or n (%).

FFR, fractional flow reserve; LAD, left anterior descending; LM, left main; NA, not available; PCI, percutaneous coronary intervention; QFR, quantitative flow ratio; RCA, right coronary artery; RCx, right circumflex.

Sensitivity analysis demonstrated that heterogeneity was predominantly present in the study of Pijls et al,^6^ which had a relatively short follow-up time of 6 months. After excluding the study from Pijls et al^6^ heterogeneity was reduced without significantly influencing the results (HR, 1.77; 95% CI, 1.23-2.53). Although a limited amount of studies is included, visual inspection of the funnel plot might suggest publication bias (Supplemental Figure S1).

### Post-PCI iFR

Data on post-PCI iFR and the long-term outcome was only reported in 1 study (Figure 1).^16^ The DEFINE PCI Trial reported data on MACE. A summary of the study characteristics is provided in Table 1, including the ratio between acute and chronic coronary syndromes and the cutoff value for impaired post-PCI physiological assessment. The study included 467 patients. The follow-up period was 1 year. Patient characteristics are summarized in Table 2. MACE was reported in 19 (4.2%) patients. The ratio between absolute numbers of adverse cardiac events and impaired post-PCI iFR is depicted in Figure 2. The analysis of the data showed a significant association between the MACE rate over the 1-year follow-up and post-PCI iFR with a cutoff value of 0.95 (HR, 3.38; 95% CI, 0.99-11.6; *P* = .04).

### Post-PCI QFR

**Figure 4:**
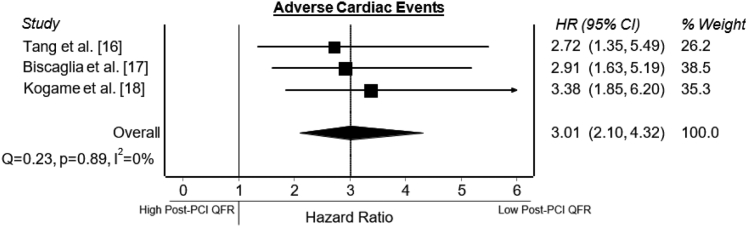
**Post-PCI QFR and adverse cardiac events.** Forest plots of hazard ratios (HRs) of post-PCI QFR and adverse cardiac events, including VOCE, defined according to the included studies. Markers represent point estimates of HRs. Marker size represents study weight. Horizontal bars indicate 95% CIs. PCI, percutaneous coronary intervention; QFR: quantitative flow ratio; VOCE, vessel-orientated cardiac events.

A total of 5 articles were identified reporting data on post-PCI QFR and long-term outcomes (Supplemental Table S3). Two studies were excluded because no sufficient data was provided or the data was incomplete. Three studies^17^, ^18^, ^19^ were sufficient to be included in the meta-analysis (Figure 1). Study quality assessment demonstrated good quality for all studies (Supplemental Table S2). All 3 studies reported on VOCE. An overview of the studies is provided in Table 1, including the ratio between acute and chronic coronary syndromes and the cutoff value for impaired post-PCI physiological assessment. The studies included 186 to 602 patients (1181 patients in total). The median follow-up period was 24 months (21–24 months). Patient and procedural characteristics were reported in Table 2 and Table 3. The prevalence of VOCE over the 3 studies varied from 6.7% to 9.4%. The ratio between absolute numbers of adverse cardiac events and impaired post-PCI QFR is depicted in Figure 2. The meta-analysis showed a significant association between an impaired post-PCI QFR and the incidence of VOCE (HR, 3.01; 95% CI, 2.10-4.32) (Figure 4). The heterogeneity of the studies was 0%. This suggests that there is no between-study heterogeneity of importance.^15^ Funnel plot did not suggest publication bias, although the amount of studies included is limited (Supplemental Figure S2).

### Post-PCI physiological assessment

A meta-analysis was performed on post-PCI physiological assessment, using a combination of the data of post-PCI FFR, iFR, and QFR. Eleven studies were included with a total of 5665 patients. The meta-analysis showed a significant association between an impaired post-PCI physiological assessment, combining all modalities, and the primary end point (HR, 2.32; 95% CI, 1.71-3.16) (Central Illustration). Heterogeneity between the studies was 68%, suggesting a moderate to substantial heterogeneity.^15^

**Central Illustration:**
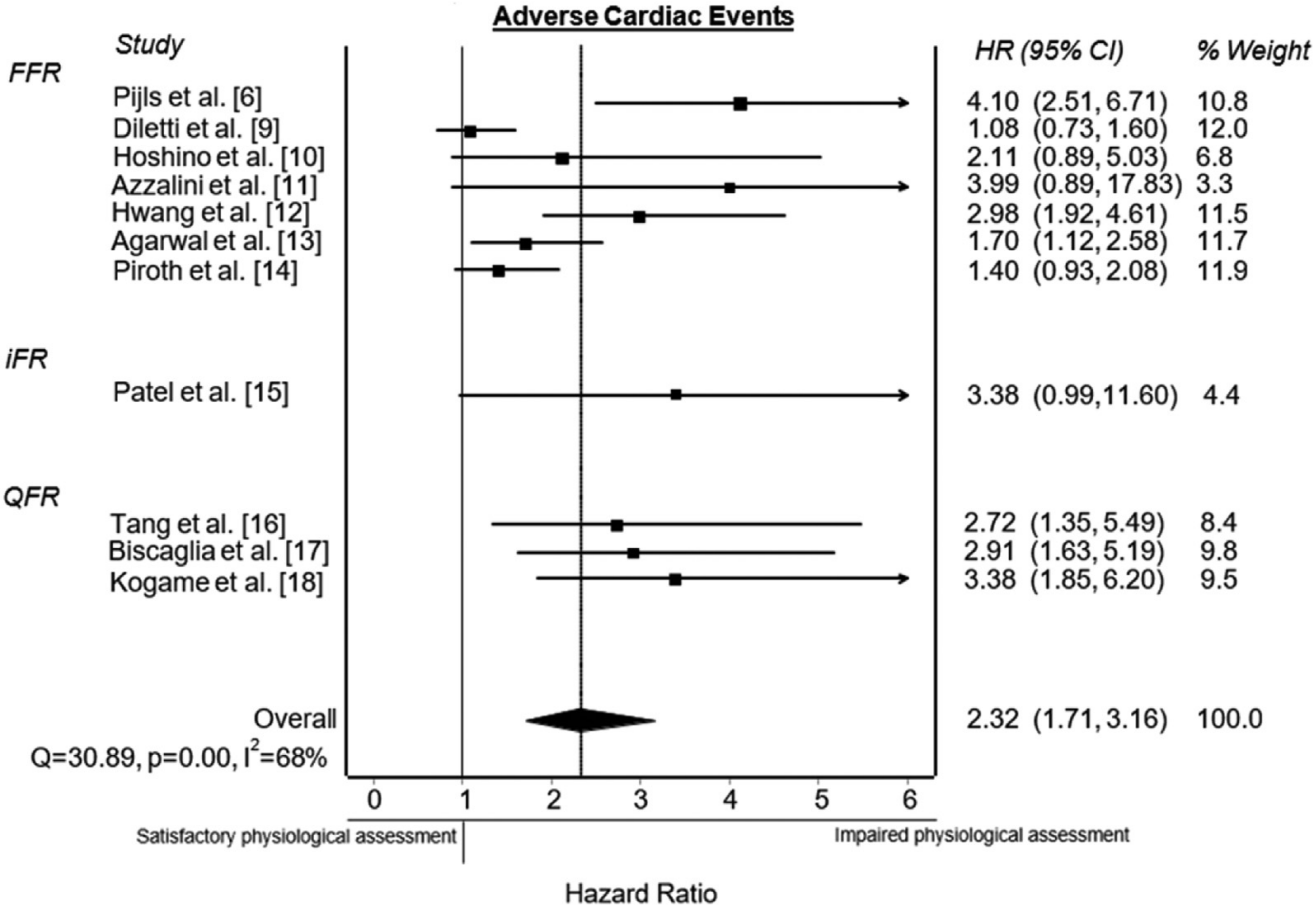
**Post-PCI physiological assessment and adverse cardiac events.** Forest plots of hazard ratios (HR) of post-PCI physiological assessment and adverse cardiac events, including MACE, VOCE, and TVF, defined according to the included studies. Markers represent point estimates of HRs. Marker size represents study weight. Horizontal bars indicate 95% CIs. FFR, fractional flow reserve; iFR, instantaneous wave-free ratio; MACE, major adverse cardiovascular events; PCI, percutaneous coronary intervention; QFR, quantitative flow ratio; TVF, target vessel failure; VOCE, vessel-orientated cardiac events.

**Figure 5:**
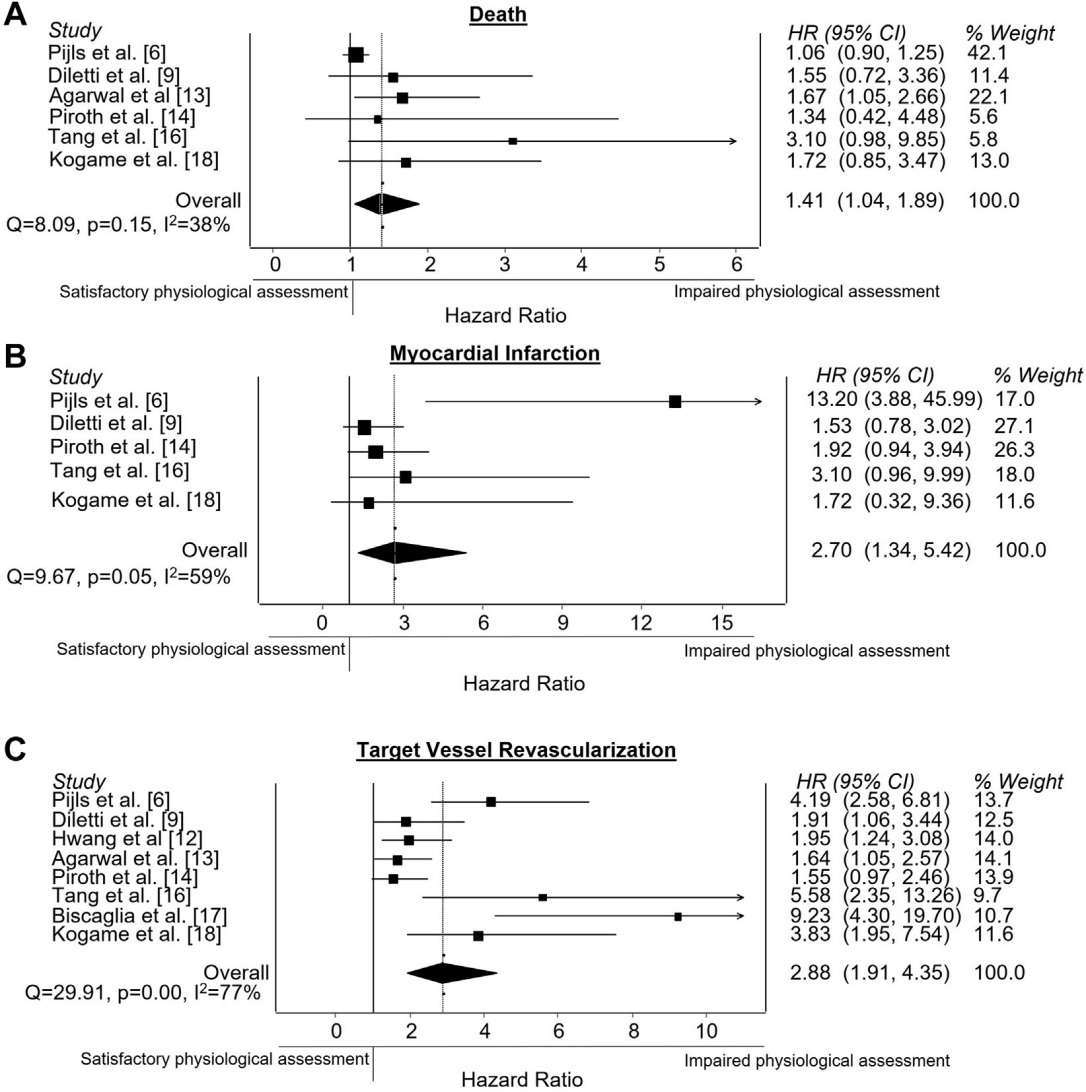
**Post-PCI physiological assessment and secondary end points.** Forest plots of hazard ratios (HRs) of post-PCI physiological assessment and (**A**) death, (**B**) myocardial infarction, and (**C**) target vessel revascularization. Markers represent point estimates of HRs. Marker size represents study weight. Horizontal bars indicate 95% CIs. PCI, percutaneous coronary intervention.

The overall incidence of death was reported by 6 studies,^6^^,^^9^^,^^13^^,^^14^^,^^17^^,^^19^ including 3495 patients. Impaired post-PCI physiological assessment was associated with an increased incidence of death (HR, 1.41; 95% CI, 1.04-1.89) (Figure 5A). Between-study heterogeneity was 38%, suggesting no significant heterogeneity of importance.^15^

Incidence of MI was reported by 5 studies,^6^^,^^9^^,^^14^^,^^17^^,^^19^ including 2921 patients. Analysis showed a significant association between impaired post-PCI physiological assessment and an increased incidence of MI (HR, 2.70; 95% CI, 1.34-5.42) (Figure 5B). Heterogeneity between the studies was 59%, suggesting moderate heterogeneity.^15^

Eight studies^6^^,^^9^^,^^12^, ^13^, ^14^^,^^17^, ^18^, ^19^ reported the incidence of TVR, including 4932 patients. Impaired post-PCI physiological assessment was associated with an increased incidence of TVR (HR, 2.88; 95% CI, 1.91-4.35) (Figure 5C). Between-study heterogeneity was 77%, suggesting that substantial heterogeneity between the studies may be present.^15^

## Discussion

The current meta-analysis studied the relation of post-PCI physiological assessment with adverse outcomes. Results demonstrate that patients with impaired post-PCI physiological assessment combining all modalities have significantly more adverse cardiovascular events with a median follow-up of 2 years. Moreover, impaired post-PCI physiological assessment results in an increased incidence of death, MI, and TVR. However, results are not influenced by the modality applied for physiological assessment.

### Post-PCI physiology

Two prior meta-analyses have been performed, including several studies about post-PCI FFR and long-term outcomes, supporting the ideal FFR cutoff value defined as 0.90.^20^^,^^21^ Unfortunately, these meta-analyses included mainly retrospective studies. Recently, Diletti et al^9^ performed a prospective study to confirm the relationship between the FFR cutoff value and MACE, showing no significant association. More recently, new modalities, such as iFR and QFR, have been investigated to provide additional data about the importance of post-PCI coronary physiology in relation to long-term outcomes.^16^, ^17^, ^18^, ^19^^,^^22^^,^^23^ The current meta-analysis pooled the data of the currently available studies on the value of post-PCI physiological assessment using FFR, iFR, and QFR.

This is the first meta-analysis providing data about several physiological assessment modalities, including iFR and QFR. Besides the meta-analysis performed by Wolfrum et al,^21^ this meta-analysis is the only study calculating the value of physiological assessment on individual end points, including death, MI, and TVR. However, more patients are included in this study, providing data and conclusions with a higher level of significance. Moreover, unlike prior meta-analyses, calculations represent a risk incidence over the entire follow-up time because the current meta-analysis is performed on HRs. HRs are preferable to odds ratios (ORs) because of the ability to represent the risk incidence over the follow-up period of the entire study instead of a single landmark in time. HRs are not calculated on cumulative data at a defined end point. HRs allow combining studies with different (median) follow-ups, while combining ORs at 1 year with ORs at 2 years may not be obvious. Moreover, an OR can only be calculated at the minimal follow-up of all patients in the study, while an HR can be calculated using all the follow-ups of all patients. The analysis in this study revealed an association between impaired post-PCI physiological assessment with the incidence of adverse cardiovascular events within 2 years. Moreover, impaired post-PCI physiological assessment is not only associated with an increased incidence of TVR but also with MI and death. Further analysis of the individual end point death should be performed by assessing specific patient characteristics, including the extent and complexity of the coronary artery disease, to enhance the clinical relevance of these findings. Nevertheless, it is unclear if optimizing the PCI result improves patient outcomes.

### Causes of suboptimal physiology

Numerous vessel-related causes can result in an impaired post-PCI physiological assessment. The residual post-PCI pressure gradient is mainly caused by the presence of residual stenosis, which also includes diffuse coronary artery disease proximal and distal of the stent. In addition, a residual focal pressure gradient can be present within the stent.^24^ Biscaglia et al^18^ investigated the location of residual stenosis post-PCI. Significant residual stenosis was present in 16% of the investigated vessels. In 87% of these vessels, the residual significant stenosis was either proximal or distal of the stent. In 13% of the vessels, there was significant residual stenosis at the location of the stent. Whereas focal residual stenosis proximal or distal of the stent often requires additional PCI, optimization of residual stenosis at the location of the stent or due to diffuse coronary artery disease proximal and distal of the stent is more difficult and may require further assessment using intracoronary imaging or pressure wire pullback curves.

### Intracoronary imaging and physiology

Intravascular coronary imaging, such as optical coherence tomography (OCT), is an important tool for decision-making prior to PCI and post-PCI.^25^ Post-PCI OCT may help to detect incomplete lesion coverage, stent malapposition, stent underexpansion, intrastent plaque protrusion, and edge dissection.^26^^,^^27^ In patients with impaired post-PCI FFR OCT was feasible for detecting suboptimal stent deployment.^26^^,^^28^ Moreover, OCT-guided optimization using postdilation resulted in higher post-PCI FFR values when compared to angiography-guided optimization of the stent result.^28^ However, it is unclear whether OCT-guided optimization of PCI leads to lower adverse cardiac events in patients with impaired post-PCI FFR. Studies are currently ongoing to assess the additional value of other intracoronary imaging-guided optimization of the PCI results in patients with post-PCI FFR <0.90.^29^

### Pressure wire pullback

Pressure wire pullbacks can be used to distinguish residual focal and diffuse coronary artery disease as well as significant residual in-stent stenosis.^30^ In addition, the Pullback Pressure Gradient Global Registry (NCT04789317) is currently ongoing to determine the impact of the Pullback Pressure Gradient Index on clinical decision-making and the impact on clinical outcomes. In residual stenosis due to diffuse coronary artery disease, the pressure wire pullback shows a gradual pressure drop over the length of the entire vessel without any visible focal stenosis on angiography.^31^ To quantify the extent of diffuse coronary artery disease, Hoshino et al^10^ recently presented a novel index, the D-index, calculated as the delta of the FFR value divided by the absolute distance between the 2 points where FFR was assessed. A higher D-index was significantly associated with MACE and VOCE.

### Limitations

This meta-analysis has a few limitations. First, publication bias could result in the overestimation of the association between impaired physiological assessment and adverse cardiac events. Non-English language studies were excluded in this meta-analysis based on practical considerations. Although study quality is assessed using the Newcastle-Ottawa Scale, the assessment of studies could be different between individual reviewers due to the design of the assessment tool. Additionally, all the studies do not have the same level of evidence (ie, studies based on retrospective data compared with prospective observational studies). Second, this is a study-level data analysis. Characteristics were unavailable at the individual patient level and could not be corrected at the individual level. Regarding study-level characteristics, variation in cutoff values for post-PCI physiological assessment is present across studies, which may explain some variation in the outcome. However, given the similarity in cut-offs, any influence on the conclusion that impaired physiological assessment post-PCI is associated with the long-term outcome is not to be expected. While the variation is not considered so large as to change the conclusion, we cannot investigate associations between a specific cutoff point for the incidence of MACE, VOCE or TVF. Third, variation in outcome definition (MACE, VOCE, or TVF) across studies is present; however, because of the overlap of components, we neither expect this to swift the conclusion of our meta-analysis. Last, analyses performed may represent moderate to substantial between-study heterogeneity. Although the measured effect of the included studies is different, they all point in the same direction.

## Conclusions

The result of this meta-analysis shows that impaired post-PCI physiological assessment is associated with increased adverse cardiac events within 2 years. Moreover, impaired post-PCI physiological assessment is not only associated with an increased incidence of TVR but also with MI and death. Therefore, prospective studies are awaited on whether physiology-based optimization of PCI results in better clinical outcomes.
