# Supplementary material for: Clinical Relevance of Impaired Physiological Assessment After Percutaneous Coronary Intervention: A Meta-analysis
Source: J Soc Cardiovasc Angiogr Interv. 2022 Sep 8;1(6):100448. doi: 10.1016/j.jscai.2022.100448 (PMC11307483; doi:10.1016/j.jscai.2022.100448)
Supplement: Supplemental Table S1 [file mmc1.docx]

**Supplementary Table 1. Description of post-PCI FFR studies**

| **First Author, Year** | **Journal** | **Country** | **Study Design** | **Population Size** | **Outcome Definition** |
| --- | --- | --- | --- | --- | --- |
| Bech, 1999 | Circulation | The Netherlands | Retrospective | 58 | Adverse cardiac events (mutually exclusive hierarchic ranking order as death, MI, unstable angina, coronary bypass surgery, repeated PTCA, and the recurrence of anginal complaints accompanied by a positive exercise test) |
| Pijls, 2002 | Circulation | United States, Europe, Asia | Prospective | 750 | MACE (composite of death, MI and TVR, either by PCI or CABG) |
| Rieber, 2002 | Zeitung für Kardiologie | Germany | Prospective | 89 | Adverse cardiac events (cardiac death, MI or TLR |
| Klauss, 2005 | Heart | Germany | Prospective | 119 | MACE (any death, MI or TVR) |
| Dupouy, 2005 | EuroIntervention | France | Prospective | 100 | MACE (death due to a cardiovascular cause, non-fatal acute MI, TVR by angioplasty or surgical coronary artery bypass, and any procedure related complication requiring a major intervention or prolonged hospital stay. |
| Stempfle, 2005 | International Journal of Cardiovascular Interventions | Germany | Retrospective | 35 | Incidence of angiographic restenosis and clinically MACE (death, MI, bypass surgery and repeat coronary intervention) |
| Jensen, 2007 | Circulation | Denmark | Prospective | 98 | Binary angiographic restenosis ≥50% |
| Ishii, 2011 | Heart and Vessels | Japan | Prospective | 33 | Restenosis (diameter stenosis more than 50% within the lesion segment) |
| Nam, 2011 | American Journal of Cardiology | South Korea | Retrospective | 80 | MACE (death, MI and TVR) |
| Leesar, 2011 | EuroIntervention | United States | Prospective | 66 | MACE (cardiac death, MI or TVR (repeat PCI or CABG)) |
| Matsuo, 2013 | Cardiovascular Intervention and Therapeutics | Japan | Prospective | 69 | Ischemic-driven TLR |
| Ito, 2014 | International Journal of Cardiology | Japan | Retrospective | 97 | MACE (composite of cardiac death, MI, stent thrombosis, and TVR) |
| Reith, 2015 | Journal of Invasive Cardiology | Germany | Prospective | 66 | MACE (any death, myocardial infarction (MI)/non-ST elevation myocardial infarction (NSTEMI) or TLR |
| Doh, 2015 | Journal of Invasive Cardiology | South Korea | Retrospective | 107 | TVF (composite of death, TVR, and non-fatal MI attributed to the target vessel) |
| Agarwal, 2016 | JACC: Cardiovascular Interventions | United States | Retrospective | 574 | MACE (defined as a composite of death, myocardial infarction and TVR) |
| Baranauskas, 2016 | EuroIntervention | Lithuania | Prospective | 74 | Restenosis (FFR-measurement < 0.80) |
| Kasula, 2016 | Heart | United States | Retrospective | 579 | MACE (composite of MI, TVR and death) |
| Li, 2017 | JACC: Cardiovascular Interventions | China, United States, Italy, Indonesia | Prospective | 1476 | TVF (cardiac death, TV-MI and TVR) |
| Piroth, 2017 | Circulation: Cardiovascular Interventions | Europe, United States, Canada | Prospective | 639 | VOCE (composite of vessel-related cardiovascular death, vessel-related spontaneous MI, and ischemia-driven TVR) |
| Pyxaras, 2018 | International Journal of Cardiovascular Imaging | Europe, China | Prospective | 26 | Suboptimal FFR |
| Lee, 2018 | JACC: Cardiovascular Interventions | South Korea, Japan | Prospective | 621 | TVF (composite of cardiac death, TV-MI and clinically driven TVR) |
| Fournier, 2019 | JAMA Cardiology | Europe, United States | Retrospective | 639 | VOCE (composite of cardiac death, TV-MI and TVR) |
| Van Bommel, 2019 | Circulation: Cardiovascular Interventions | The Netherlands | Prospective | 959 | Composite of cardiac death, non-fatal MI, or TVR |
| Lee, 2019 | JACC: Cardiovascular Interventions | South Korea | Retrospective | 83 | TLF (composite of death from cardiac cause, TV-MI, TLR) |
| Chung, 2019 | International Journal of Cardiovascular Imaging | South Korea | Prospective | 151 | MACE (cardiac death, target vessel related myocardial infarction (MI) and clinically driven TVR |
| Azzalini, 2019 | Journal of Invasive Cardiology | Italy | Prospective | 65 | MACE (composite of all-cause death, non-fatal MI and TVR) |
| Hwang, 2019 | EuroIntervention | South Korea, Japan | Prospective | 835 | TVF (composite of cardiac death, TV-MI, clinically driven TVR) |
| Hoshino, 2019 | EuroIntervention | Japan | Prospective | 201 | MACE (VOCE (cardiovascular death, vessel-related spontaneous myocardial infarction, ischemia-driven LAD revascularization) and non-target VOCE) |
| Hakeem, 2019 | JACC: Cardiovascular Interventions | United States | Retrospective | 574 | MACE (composite of death, MI (not related to intervention) and TVR). |
| Hwang, 2020 | JACC: Cardiovascular Interventions | South Korea, China, Japan | Prospective | 2200 | TVF (composite of cardiac death, TV-MI, clinically driven TVR) |
| Hamaya, 2020 | JAMA Network Open | South Korea, Japan, China, United States | Prospective | 1488 | TVF (composite of death from cardiovascular causes, TV-MI and clinically driven TVR |
| Diletti, 2021 | Circulation: Cardiovascular Interventions | The Netherlands | Prospective | 959 | MACE (composite of cardiac death, MI, any revascularization) |

Legend: FFR: Fractional Flow Reserve, MACE: Major Adverse Cardiac Events, MI: Myocardial Infarction, PTCA: Percutaneous Transluminal Coronary Angioplasty, TLF: Target Lesion Failure, TLR: Target Lesion Revascularization, TVF: Target Vessel Failure, TV-MI: Target Vessel Myocardial Infarction, TVR: Target Vessel Revascularization, VOCE: Vessel-Orientated Cardiac Events

**Supplementary Table 2. Newcastle-Ottawa Scale for Assessment of Study Quality**

| **Author and Publication Year** | **Design** | **Selection** | **Comparabiliy** | **Outcome** | **Total** |
| --- | --- | --- | --- | --- | --- |
| *Post-PCI FFR* | | | | | |
| Pijls et al., 2002 | Cohort | **** | * | *** | 8/9 |
| Diletti et al., 2021 | Cohort | **** | ** | *** | 9/9 |
| Hoshino et al., 2019 | Cohort | *** | - | *** | 6/9 |
| Azzalini et al., 2019 | Cohort | **** | - | *** | 7/9 |
| Hwang et al., 2019 | Cohort | **** | ** | *** | 9/9 |
| Agarwal et al., 2016 | Cohort | **** | ** | *** | 9/9 |
| Piroth et al., 2017 | Cohort | *** | ** | ** | 7/9 |
| *Post-PCI iFR* | | | | | |
| Patel et al., 2022 | Cohort | **** | - | *** | 7/9 |
| *Post-PCI QFR* | | | | | |
| Tang et al., 2020 | Cohort | *** | * | *** | 7/9 |
| Biscaglia et al., 2019 | Cohort | **** | ** | *** | 9/9 |
| Kogame et al., 2019 | Cohort | *** | * | *** | 7/9 |

Legend: FFR: Fractional Flow Reserve, iFR: instantaneous wave-free ratio, QFR: Quantitative Flow Ratio

**Supplementary Table 3. Description of post-PCI QFR studies**

| **First Author, Year** | **Journal** | **Country** | **Study Design** | **Population Size** | **Outcome Definition** |
| --- | --- | --- | --- | --- | --- |
| Biscaglia, 2019 | JACC: Cardiovascular Intervention | Italy, Spain | Prospective | 602 | VOCE (composite of vessel-related cardiovascular death, vessel-related MI, and ischemia-driven target vessel revascularization (TVR) |
| Kogame, 2019 | JACC: Cardiovascular Intervention | Europe | Retrospective | 440 | VOCE (composite of vessel-related cardiac death, vessel-related MI and TVR) |
| Tang, 2021 | International Journal of Cardiovascular Imaging | China | Retrospective | 186 | VOCE (composite of vessel-related cardiovascular death, vessel-related MI, and TVR) |
| Saito, 2021 | Catheterization and Cardiovascular Interventions | Europe, Brazil, Australia | Prospective | 235 | MACE (composite of cardiac death, myocardial infarction, or clinically driven TLR) |
| Erbay, 2021 | Circulation: Cardiovascular Interventions | Germany | Retrospective | 792 | MACE (composite of all-cause death, non-fatal myocardial infarction, and ischemia-driven revascularization by either PCI or CABG) |

Legend: CABG: Coronary artery bypass grafting, MACE: Major Adverse Cardiac Events, MI: Myocardial Infarction, PCI: Percutaneous Coronary Intervention, QFR: Quantative Flow Ratio, TLR: Target Lesion Revascularization, TVF: Target Vessel Failure, TV-MI: Target Vessel Myocardial Infarction, TVR: Target Vessel Revascularization, VOCE: Vessel-Orientated Cardiac Events

**Supplementary Figure 1. Funnel plot analysis for publication bias regarding FFR and Adverse Cardiac Events**

Legend: Solid vertical line represents the summary estimate of the effect of all ordinances on Adverse Cardiac Events (assuming a random effects meta-analysis), and the dashed lines represent the 95% confidence interval. HR: Hazard Ratio; FFR: Fractional Flow Reserve


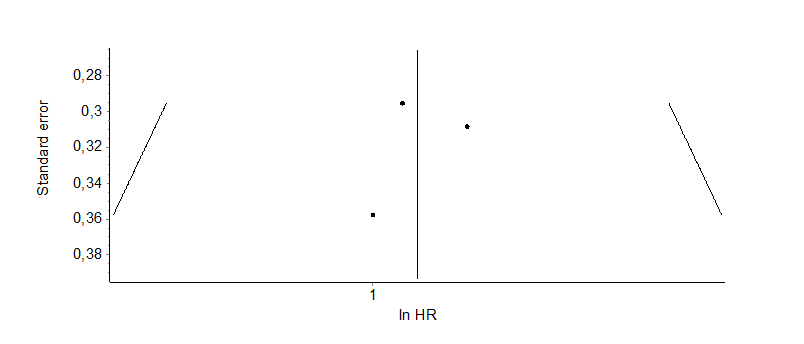


**Supplementary Figure 2. Funnel plot analysis for publication bias regarding QFR and Adverse Cardiac Events**

Legend: Solid vertical line represents the summary estimate of the effect of all ordinances on Adverse Cardiac Events (assuming a random effects meta-analysis), and the dashed lines represent the 95% confidence interval. HR: Hazard Ratio; QFR: Quantitative Flow Ratio
